# Fitness Cost of Transgenic *cry1Ab/c* Rice Under Saline-Alkaline Soil Condition

**DOI:** 10.3389/fpls.2018.01552

**Published:** 2018-10-23

**Authors:** Jianmei Fu, Xiaoling Song, Biao Liu, Yu Shi, Wenjing Shen, Zhixiang Fang, Li Zhang

**Affiliations:** ^1^Weed Research Lab, College of Life Science, Nanjing Agricultural University, Nanjing, China; ^2^Nanjing Institute of Environmental Sciences, Ministry of Environmental Protection, Nanjing, China; ^3^College of Plant Protection, Nanjing Agricultural University, Nanjing, China

**Keywords:** biosafety, *Bt*, Cry1Ab/c protein, fitness cost, saline-alkaline soil, target insect, transgenic rice

## Abstract

The environmental release and biosafety of transgenic *Bt* crops have attracted global attention. China has a large area of saline-alkali land, which is ideal for large-scale production of *Bt* transgenic rice. Therefore an understanding of the fitness of *Bt* transgenic rice in saline-alkaline soils and the ability to predict its long-term environmental effects are important for the future sustainable use of these crops. In the present study, we aimed to evaluate the fitness of *cry1Ab/c* transgenic rice in both farmland and natural ecosystems. Transgenic *cry1Ab/c* rice Huahui1, for which a national biosafety certificate was obtained, was grown on normal farmland and saline-alkaline soils in a glass greenhouse. The expression pattern of exogenous Cry1Ab/c protein, and vegetative and reproductive fitness of rice were assessed. The expression of the exogenous Cry1Ab/c protein in the transgenic rice grown on saline-alkaline soil was lower than that in the strain grown on farmland soil. Under both the soil conditions, vegetative growth abilities, as evaluated by tiller number and biomass, and reproductive growth abilities, as measured by filled grain number and filled grain weight per plant, showed a significantly higher fitness cost for Huahui1 than that for the parental rice Minghui63 grown under the same soil conditions. In saline-alkaline soil, the fitness cost of Huahui1was moderately higher than that of Minghui63. Therefore, the ecological risk of *cry1Ab/c* transgenic rice is not expected to be higher than that of parental rice Minghui63 if the former escapes into natural saline-alkaline soil. The results of the present study provide a scientific basis to improve environmental safety assessment of the insect-resistant transgenic rice strain Huahui1 before commercialization.

## Introduction

In 2016, the global planting area of genetically modified crops reached 185.1 million ha. Genetically modified crops planting areas, nations, species, growth rates, and other parameters have substantially improved since 1996, and have realized significant economic, environmental, and social benefits (http://www.isaaa.org/). Rice (*Oryza sativa*) is a staple food for more than half the world's population and therefore plays an important role in the management of global food security. A major group of lepidopteron rice insects has caused serious rice production losses in China. Therefore, the *Bt* gene from *Bacillus thuringiensis*, which encodes an insecticidal crystalline protein, was introduced into rice. This has become a major strategy for the development of transgenic rice in China (Tu et al., 2000; High et al., 2004). During the past 20 years, numerous insect resistance genes have been identified, some of which have anti-lepidopteron activity (*cry1Aa, cry1Ab, cry1Ac, cry1Ab/Ac, cry1C, cry2A*, and *CpT1*), whereas others exhibit anti-helipterum activity (*Galanthus nivalis agglutinin, gna*, and *Pinellia ternata agglutinin, pta*). These genes have been introduced into rice to develop insect-resistant transgenic lines (Shu et al., 1998, 2000; Tan et al., 2011; Wang et al., 2012a,b; Jiang et al., 2013b; Jian et al., 2014). Currently, most insect-resistant transgenic rice lines in China are in the field trial stage (Tang et al., 2006; Wang et al., 2012a,b). Experimental results have indicated that transgenic rice effectively resists lepidopteron damage in field and reduces environmental and crop contamination by reducing pesticide application rates. It is anticipated that these transgenic varieties will have relatively higher commercial value (Tu et al., 1998; Shu et al., 2002; Ye et al., 2003).

Biosafety is an important factor limiting the commercialization of these transgenic rice lines, and it encompasses both environmental and food safety. On October 22, 2009, the Ministry of Agriculture of China issued two biosafety certificates for the production and application of the insect-resistant transgenic rice lines Huahui-1 (HH1) and *Bt* Shanyou-63 (Bt-SY63) in Hubei Province (Wang et al., 2010; Xia et al., 2010; Liu et al., 2015; Zhang et al., 2016). These permits were renewed in December 2014 but to date the crops have not yet been commercialized. In January 2018, the US FDA completed a consultation on the safety assessment of HH1 insect-resistant transgenic rice line. It was concluded that in terms of nutritional value, safety, and other criteria of human food and animal feed derived from HH1 rice, there were no significant differences compared with those derived from commercial rice (http://news.sciencenet.cn/). Therefore, the commercial production and application of transgenic rice should be expedited.

Environmental risk assessment of insect-resistant transgenic rice must be rigorous and include evaluations of weediness (Xia et al., 2011; Jiang et al., 2016), foreign gene excursions (Chen et al., 2004; Xia et al., 2016), hybrid progeny fitness effects (Yang et al., 2015) and other factors. It must be determined whether the escaped exogenous genes can survive in the local natural ecosystem or radiate beyond it. The fitness of transgenic rice in farmland and natural ecosystems must also be established. These are the main criteria to assess and monitor environmental safety of transgenic rice. If HH1 is produced on a large scale, it might persist in farmland ecosystem via seed spraying or transgene introgression into weedy rice and other populations. It might also reach common wild rice population via the same routes and have access to waste lots, saline-alkaline land and other sites outside the farmland (Jenczewski et al., 2003). Therefore, it could propagate in the natural ecosystem with unpredictable ecological consequences (Yang et al., 2015; Lu et al., 2016).

Another potential problem associated with the use of these crops is a possible change in the fitness of *Bt* transgenic rice under various environmental conditions, as reported by several studies (Wang et al., 2012a,b; Jiang et al., 2013b; Li et al., 2013; Jian et al., 2014). In particular, different insect pressure conditions vary in terms of their fitness effects on vegetative and reproductive growth abilities of transgenic rice. Tu et al. (2000) reported that under conditions of normal insect pressure in field, the yield of *cry1Ab/c* transgenic rice Bt-SY63 was 28.9% higher than those of the parental rice SY63, in contrast, the plant height, tiller number, grain number per panicle, and other parameters of Bt-SY63 were not significantly higher than those of parental SY63. Other studies have reported that *cry1Ab/c, cry1C*, and *cry2A* transgenic rice presented with significant yield advantages over parental rice under severe natural insect pressure in field (Tang et al., 2006; Xia et al., 2011; Jiang et al., 2014, 2016). Dong et al. (2017) reported that the vegetative and reproductive growth abilities of crop-wild type hybrid progeny harboring the *Bt/CpT1* gene were significantly higher than those of the wild type parental rice under severe field natural insect pressure. Yang et al. (2015) showed that the vegetative and reproductive growth abilities of the F_4_-F_7_-weed hybrid progeny harboring the *Bt/CpT1* gene were significantly higher than those of the parental crop-weed hybrid rice under severe field natural insect pressure. It is evident that the exogenous *Bt* gene confers significant fitness benefits to transgenic, wild type and weedy rice harboring the gene under target insect pressure selection in field. However, Chen et al. (2006) and Xia et al. (2010) reported that the total yield of *Bt/CpT1* transgenic rice was significantly lower than that of the parental rice MH86 under low insect pressure. Other studies have reported that under relatively low insect pressure conditions, transgenic rice with the exogenous *Bt* gene usually have an obvious yield disadvantage compared with that of the parental rice (Shu et al., 2002; Kim et al., 2008; Jiang et al., 2013b).

In contrast to the farmland condition, transgenic rice might exhibit different fitness effects in natural ecosystems having comparatively lower target insect pressure and water quality, inferior soil fertility, and higher weed competition. Su et al. (2013) found that the reproductive ability of *cry1Ab* transgenic rice did not significantly differ from that of the parental MH86 rice when the natural insect pressure was low under simulated semi-wild cultivation conditions. There were also no significant differences between *cry1Ab/c* transgenic rice and parental MH63 rice in terms of reproductive and competitive abilities in a natural ecosystem simulating high insect pressure (Liu et al., 2015).

Another factor that considerably affects crop vegetative and reproductive growth abilities is soil salinization. The global area of saline-alkaline land is ~9.5 × 10^9^ hm^2^ (Wild, 2004). In China, the area of saline-alkaline land is ~3.6 × 10^7^ hm^2^, and it is mainly distributed in Northeastern, Northwestern, and Northern China, certain coastal areas, and others (Yang, 2008). These regions are also the main rice producing areas in China (Jiang et al., 2013a). In the future, *cry1Ab/c* transgenic rice could be commercially cultivated in a large area in China. It might be sown on farmlands with high salinity. Some seeds might escape and disperse into regions with natural saline-alkaline soil surrounding the farmlands. However, to date, there have been no reports on the fitness performance of transgenic rice derived from the seeds that escaped from the tillage system and those that dispersed into natural saline-alkaline soils without target insect pressure.

From the perspective of biosafety and ecological security, it is necessary to study the vegetative and reproductive growth and long term environmental behavior of *cry1Ab/c* transgenic HH1 rice that is dispersed into regions with saline-alkaline soils and relatively low insect pressure. The available area of cultivated and arable lands is limited in China. Therefore, it is important to develop and utilize the saline-alkaline soil for rice production, especially in coastal beach areas (Chen et al., 2018a). The vegetative and reproductive growth abilities of *cry1Ab/c* transgenic HH1 rice under saline-alkaline soil conditions should be better understood in light of its potential use as an insect-resistant transgenic rice strain in China. In the present study, we aimed to (1) evaluate the fitness of *cry1Ab/c* transgenic rice in saline-alkaline soil, and the farmland soil as a control and (2) identify the potential environmental risks associated with its commercial production under both farmland and saline-alkaline soil in a glass greenhouse. The expression of Cry1Ab/c protein was measured at different growth stages and in various tissues of *cry1Ab/c* transgenic rice. Furthermore, the vegetative and reproductive growth abilities of *cry1Ab/c* transgenic rice were investigated. Finally, the fitness effects of *cry1Ab/c* transgenic HH1 and parental MH63 rice under the two different soil conditions were analyzed to provide a theoretical basis for evaluating the environmental safety of insect-resistant *cry1Ab/c* transgenic rice.

## Materials and methods

### Rice

The *cry1Ab/c* transgenic rice Huahui-1 (HH1) is an indica rice strain. Biosafety certificates have been issued for its commercial production in Hubei Province, China. Its non-transgenic counterpart, Minghui-63 (MH63), is an elite indica cytoplasmic male sterility restorer line. The seeds were provided by the Huazhong Agricultural University, Wuhan, China for use in the present study. The *cry1Ab/c* transgene was synthesized from the 1,344 bp *cry1Ab* gene (GeneBank accession no. X54939) and the 486 bp *cry1Ac* gene (GeneBank accession no. Y09787), and driven by the rice actin1 promoter (Tu et al., 1998). Huahui-1 presented a high level of δ-endotoxin expression and was identified to possess insecticidal activity against stem borers (*Chilo suppressalis* and *Tryporyza incertulas*) and leaf borers (*Cnaphalocrocis medinalis*) (Tu et al., 2000).

### Soil

Saline-alkaline soil (topsoil, 0–30 cm layer) was collected from a typical saline-alkaline region in the surrounding coastal wetland near the National Nature Reserve, Rare Birds in Yancheng, Jiangsu, China (32°48′47″-34°29′28″ N, 119°53′45″-121°18′12″ E). The control was farmland soil (topsoil, 0–30 cm layer) from a paddy field located in Luhe District, Nanjing, Jiangsu, China (32°11′-32°27′ N, 118°34′-119°03′ E). The physicochemical properties of the farmland soil were determined after mixing it with nutrient soil (1:1) as potting soil and before sowing rice. A comparison of the physicochemical properties of the two soils is presented in Table 1. The total nitrogen, total phosphorus, available phosphorus, and available potassium content were all significantly lower in the saline-alkaline soil than in the farmland soil. The salt content (Luo et al., 2017) and pH were significantly higher in the saline-alkaline soil than in the farmland soil. The treatment soil was considered to have a moderate to high saline-alkaline content relative to the salinity grade of coastal saline-alkaline soil (Dong et al., 2012). In addition, the organic matter content was significantly higher in the saline-alkaline soil than in the farmland soil.

**Table 1:** Physical and chemical properties of the two soils.

**Physical and chemical properties of soil**	**Farmland soil**	**Saline-alkali soil**
Salt content (%)	0.03 ± 0.00**	0.38 ± 0.00
Organic matter (g/kg)	6.59 ± 0.46**	46.28 ± 3.31
Total nitrogen content (g/kg)	3.02 ± 0.09**	0.35 ± 0.04
Total phosphorus content (g/kg)	1.47 ± 0.09**	0.52 ± 0.03
Total potassium content (g/kg)	9.59 ± 0.16	9.79 ± 0.2
Available phosphorus content (mg/kg)	11.62 ± 2.16**	4.70 ± 0.54
Available potassium content (mg/kg)	973.00 ± 13.67**	90.36 ± 2.81
pH	7.32 ± 0.04**	9.03 ± 0.05

Farmland soil values with ** were significantly different from those of saline-alkaline soil according to the t-test (p < 0.01).

### Experimental design

A pot experiment was set up in the glasshouse of the Nanjing Environmental Science Institute of the Ministry of Environmental Protection of China. The test site is surrounded by residential areas and office buildings. There is no farmland within 5 km of this facility. No rice, vegetable, or other crops were planted in the vicinity to ensure that the test was conducted under environmentally secure conditions with very low insect pressure and minimal risk of target insect attack. The fitness effects of transgenic rice with the *cry1Ab/c* gene were evaluated by simulating saline-alkaline and farmland soil conditions. Rice seeds were surface-sterilized in 75% *v*/*v* alcohol, washed three times with ultrapure water, and stored in a humid incubator at 30°C until germination. The seedlings were temporarily grown on a small culture plate. At the five-leaf stage, the seedlings were transplanted into large pots (840 mm length × 560 mm width × 360 mm height) filled with either saline-alkaline or farmland soil. To avoid possible microsite effects, 10 replicate pots were randomly distributed. Each replicate consisted of 12 individuals arranged in 4 rows and 3 columns at 19–21 cm intervals. The plants in farmland soil were watered frequently and not treated with insecticides at any point in their life cycle. Weeds in the pots were manually removed. The plants in saline-alkaline soil were watered frequently. The water layer was maintained at 1 cm depth above the soil surface to ensure a constant salt content. No agricultural maintenance was carried out. All remaining test materials were burned after completion of the trial.

### Insect pressure in the experiments

MH63 is a parental rice strain lacking an insect resistance gene. It was used to determine the target insect pressure index (%). The assessments were conducted at the jointing and heading stages, as rice borer attacks are most likely to occur at these stages. In addition, stem borers (*C. suppressalis* and *T. incertulas*) can cause dead heart and white spike, and leaf borers (*C. medinalis*) can induce leaf rolling. The dead heart rate was calculated from the number of dead hearts divided by the number of tillers per plant. The leaf rolling rate was determined by the number of rolled leaves divided by the total number of leaves per plant or the total number of tillers multiplied by three. Finally, the target insect pressure index was obtained as the sum of dead heart rate and leaf rolling rate.

In the present study, only a few non-target spiders (*Arachnida*) and insects (ladybugs [*Coccinellidae*] and locusts [*Locusta migratoria manilensis*]) were observed. No target insects of *Bt* transgenic rice such as rice stem borers (*Scirpophaga incertulas, C. suppressalis*, and *Sesamia inferens*) or rice leaf borers (*C. medinalis*) were detected. Dead heart and leaf rolling caused by the aforementioned target insects were not observed in HH1 or MH63 rice, suggesting that the target insect pressure was very low for all rice material tested under the growth conditions of the present study.

### Quantification of Cry1Ab/c protein by enzyme-linked immunosorbent assay

Rice leaves and stems were collected at tilling (July 20, 2017), jointing (August 5, 2017), heading (September 5, 2017), filling (September 25, 2017), and maturing (October 25, 2017). The samples were immersed in liquid nitrogen upon collection. They were stored at −80°C in an ultralow-temperature freezer. To minimize differences in Cry1Ab/c protein expression due to environmental factors, five individual samples per plot were pooled and five replicate pots were randomized. The expression of Cry1Ab/c protein in HH1 rice was quantified using a QualiPlate Kit for Cry1Ab/Cry1Ac (EnviroLogix Inc., Portland, ME, USA). About Twenty milligrams of tissue sample were weighed and its mass was recorded. The sample was pulverized in liquid nitrogen with a tissue crusher (TissueLyser II; Qiagen, Hilden, Germany). One milliliter Cry1Ab/c 1 × extraction buffer (EnviroLogix Inc.) and the milled sample were added to 2 mL microreaction tubes. The samples were incubated at 4°C on a shaker for 30 min at 150 rpm. The sample was then centrifuged at 10,000 × *g* for 5 min at 4°C. The supernatant was collected and diluted to 50–200-fold with 1 × extraction buffer. The Cry1Ab/c protein was quantified according to the instructions provided by the manufacturers of the corresponding QualiPlate Kits for Cry1Ab/Cry1Ac. Cry1Ab/c was quantified according to the method of Zhang et al. (2016). The optical density (OD) was measured at 450 nm using a Microplate Reader (Infinite M2000; Tecan Group Inc., Männedorf, Switzerland). The reader was calibrated by plotting a standard curve of OD against protein content using the following ranges of Bt protein (Cry1Ab) standard: 0.03125, 0.0625, 0.125, 0.25, 0.5, 1.0, 2.0, and 4.0 ng ml^−1^. The level of Cry1Ab/c protein in the fresh rice samples was determined using a standard curve and the dilution ratios of the extraction solution (μg g^−1^ FW). The Cry1Ab standards were prepared from purified Cry1Ab protein (EnviroLogix Inc).

### Measurement of fitness indices on vegetative and reproductive growth

To determine the morphological traits of the rice plants during vegetative growth, the number of tillers and shoot height at tilling, heading, filling, and maturing stages of 20 randomly selected plants were measured. Border rows were excluded to avoid edge effects. The samples were collected by excision at the soil surface. They were oven-dried at 80°C to a constant weight for biomass determination at tilling, heading, filling, and maturing stages.

To determine the morphological traits at the maturing stages of HH1 and MH63 rice, the following parameters were measured: effective number of panicles per plant (>5 filled grains), panicle length, panicle weight (weight of effective number of panicles per plant), filled grain number per plant, filled grain weight per plant, total grain number per plant (filled grain number per plant plus empty grain number per plant), filled grain number per panicle (filled grain number per plant divided by effective number of panicles per plant), 1,000-grain weight (weight of 1,000 randomly selected grains), and seed setting rate (ratio of the number of filled grains per plant to the total number of grains per plant).

To evaluate the net cost or benefits of vegetative and reproductive growth ability, according to the method of Song et al. (2004), the average of each traits of MH63 rice was defined as “1,” and the average values of each HH1 compared with those of the MH63 control was defined as fitness, and calculated the total fitness. Fitness related to each stage was calculated as the mean of all traits within this stage, the whole life-history fitness was the mean of the four stages. Independent *t*-tests were used to analyze the fitness differences.

### Data collection and analysis

The expression of exogenous Cry1Ab/c protein in HH1 rice might be affected by relative differences between the farmland and saline-alkaline soils. Therefore, independent *t*-tests were run to identify any significant differences between the farmland and saline-alkaline soil treatments in terms of expression of exogenous Cry1Ab/c protein in each tissue and at each growth stage. Duncan's multiple comparison tests was used to analyze the temporal and spatial dynamics of Cry1Ab/c expression at different growth stages and in tissues under each soil condition. The three-way analysis of variance (ANOVA) was used to estimate the impact of soil conditions (two), growth stage (five), and tissue (two) on Cry1Ab/c protein expression.

Exogenous gene insertion might influence the fitness indices of HH1 rice during the vegetative and reproductive growth stages. Therefore, relative differences between HH1 and MH63 rice in terms of vegetative and reproductive traits were compared using independent *t*-test. The three-way ANOVA was used to estimate the effect of soil conditions (two), rice lines (two), and growth stages (four) on vegetative performance. The two-way ANOVA was used to estimate the effect of soil conditions (farmland and saline-alkaline) and rice lines (HH1 and MH63) on reproductive performance. All statistical analyses were performed using SPSS v. 16.0 for Windows (IBM Corp., Armonk, NY, USA).

## Results

### Saline-alkali stress significantly reduced bt expression in transgenic line

**Figure 1:**
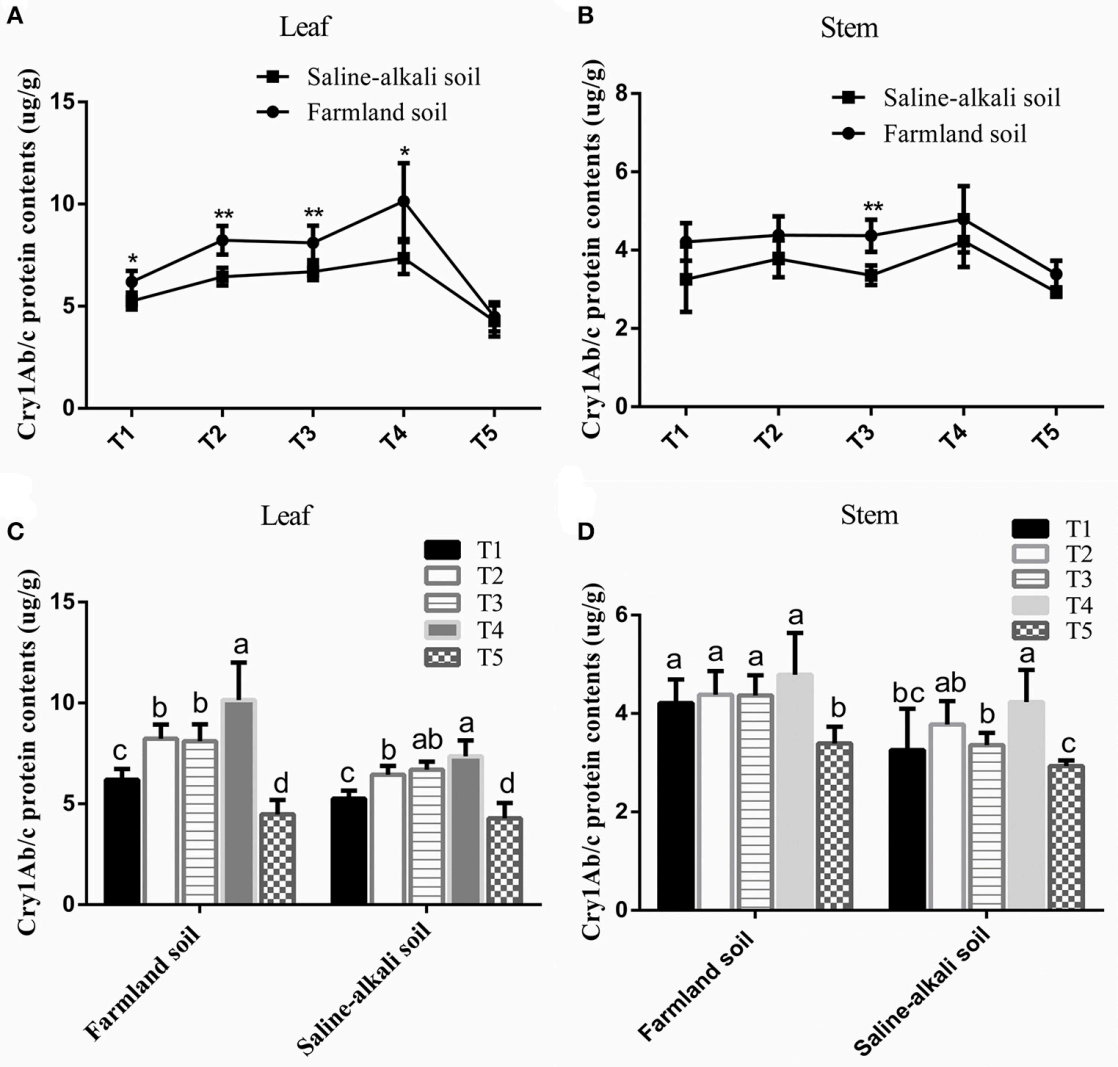
Cry1Ab/c protein level in the leaf and stem of HH1 rice grown in simulated farmland and saline-alkaline soils at five stages (T1: tillering, T2: jointing, T3: heading, T4: filling, and T5: maturing stages). Farmland soil values with * and ** were significantly different from those for saline-alkaline soil according to the *t*-test (*p* < 0.05 and *p* < 0.01, respectively) in the leaf **(A)** and stem **(B)**. a, b, c, and d indicate significant differences between the five growth stages of HH1 rice grown on the same soil according to Duncan's multiple range test (*p* < 0.05) in the leaf **(C)** and stem **(D)**.

As shown in Figure 1, the expression level of Cry1Ab/c protein in the leaves and stems of HH1 rice in farmland soil was higher than that of the plants at the same growth stage in saline-alkaline soil. Except at the tillering stage, the expression level of Cry1Ab/c in the leaves significantly differed between the two soil conditions (Figure 1A) (*p* < 0.05). In the stems, the expression level of Cry1Ab/c significantly differed between farmland and saline-alkaline soils only at the heading stage (Figure 1B) (*p* < 0.05).

The expression of Cry1Ab/c in transgenic HH1 rice varied temporally and spatially at different growth stages under the same soil conditions. It was more than two times higher in the leaves than in the stems at most growth stages. In both soil types, foliar Cry1Ab/c protein expression initially increased, and then decreased with growth. It reached the maximal levels of 10.15 μg g^−1^ FW (farmland) and 7.36 μg g^−1^ FW (saline-alkaline) at the filling stage, but significantly decreased to 4.48 μg g^−1^ FW (farmland) and 4.28 μg g^−1^ FW (saline-alkaline) at the maturing stage (Figure 1C). In the stem, Cry1Ab/c expression varied substantially between the soil types. In farmland soil, Cry1Ab/c expression did not significantly differ among the tillering, jointing, heading, and filling stages (*p* > 0.05). It reached the maximal level of 4.79 μg g^−1^ FW at the filling stage and significantly decreased to 4.48 μg g^−1^ FW at the maturing stage. In saline-alkaline soil, Cry1Ab/c expression level significantly differed among the tillering, heading, and filling stages (*p* < 0.05). It reached the maximal level of 4.23 μg g^−1^ FW at the filling stage and significantly decreased to 3.39 μg g^−1^ FW at the maturing stage (Figure 1D).

The three-way ANOVA indicated that the soil, growth stage, and tissue significantly influenced Cry1Ab/c expression. The interactions between soil and tissue and between growth stage and tissue significantly affected Cry1Ab/c expression. However, the interactions between soil and growth stage and among soil, growth stage, and tissue did not affect Cry1Ab/c expression (Table S1). However, the overall expression ranged between 2.93 μg g^−1^ FW and 10.15 μg g^−1^ FW. The exogenous Cry1Ab/c protein concentrations in transgenic rice HH1 should suffice to kill target insects such as *S. incertulas, C. suppressalis*, and *C. medinalis*.

### Effect of transgene expression on vegetative growth indices of transgenic rice HH1 under saline-alkali soil

#### Plant height

**Figure 2:**
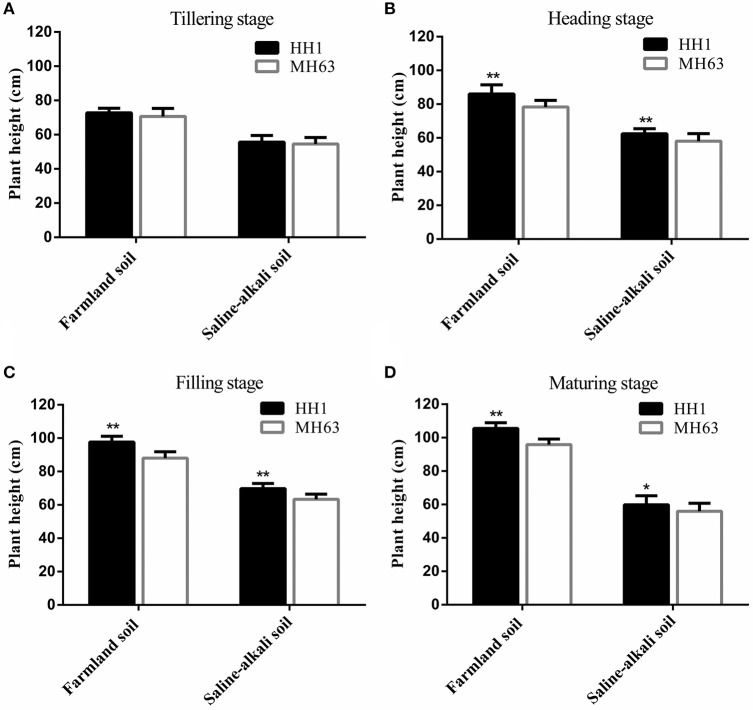
Plant height (mean ± SEM) of HH1 and MH63 rice grown on simulated farmland and saline-alkaline soil at four stages: **(A)** tilling stage, **(B)** heading stage, **(C)** filling stage, **(D)** maturing stage. Values for HH1 with * and ** are significantly different from those of MH63 according to the *t*-test (*p* < 0.05 and *p* < 0.01, respectively).

As shown in Figure 2, there were significant differences between HH1 and MH63 rice under the two soil conditions in terms of plant height (*P* < 0.01). The shoot height of the same rice line was significantly higher for plants grown in farmland than in saline-alkaline (~29.41–71.30% at the four key growth stages).

In both the soil types, the height of HH1 and MH63 was the same throughout the growth stage. It gradually increased with growth. On farmland soil, the height of HH1 plant was significantly higher than that of MH63 rice by ~10–11% at the heading, filling, and maturing stages (*p* < 0.01). On saline-alkaline soil, the height of HH1 plant was significantly higher than that of MH63 rice by ~7–10% at the heading, filling, and maturing stages (*p* < 0.01). There was a significant fitness benefit between HH1 and MH63 rice in terms of plant height grown on saline-alkaline soil. However, the magnitude of this fitness benefit was relatively smaller than that of the rice grown on farmland soil (Table 3). The three-way ANOVA indicated that the soil, exogenous gene, growth stage, interaction between exogenous gene and soil, interaction between soil and growth stage, and interaction between exogenous gene and growth stage significantly affected plant height (Table S2).

#### Effective tiller number

**Figure 3:**
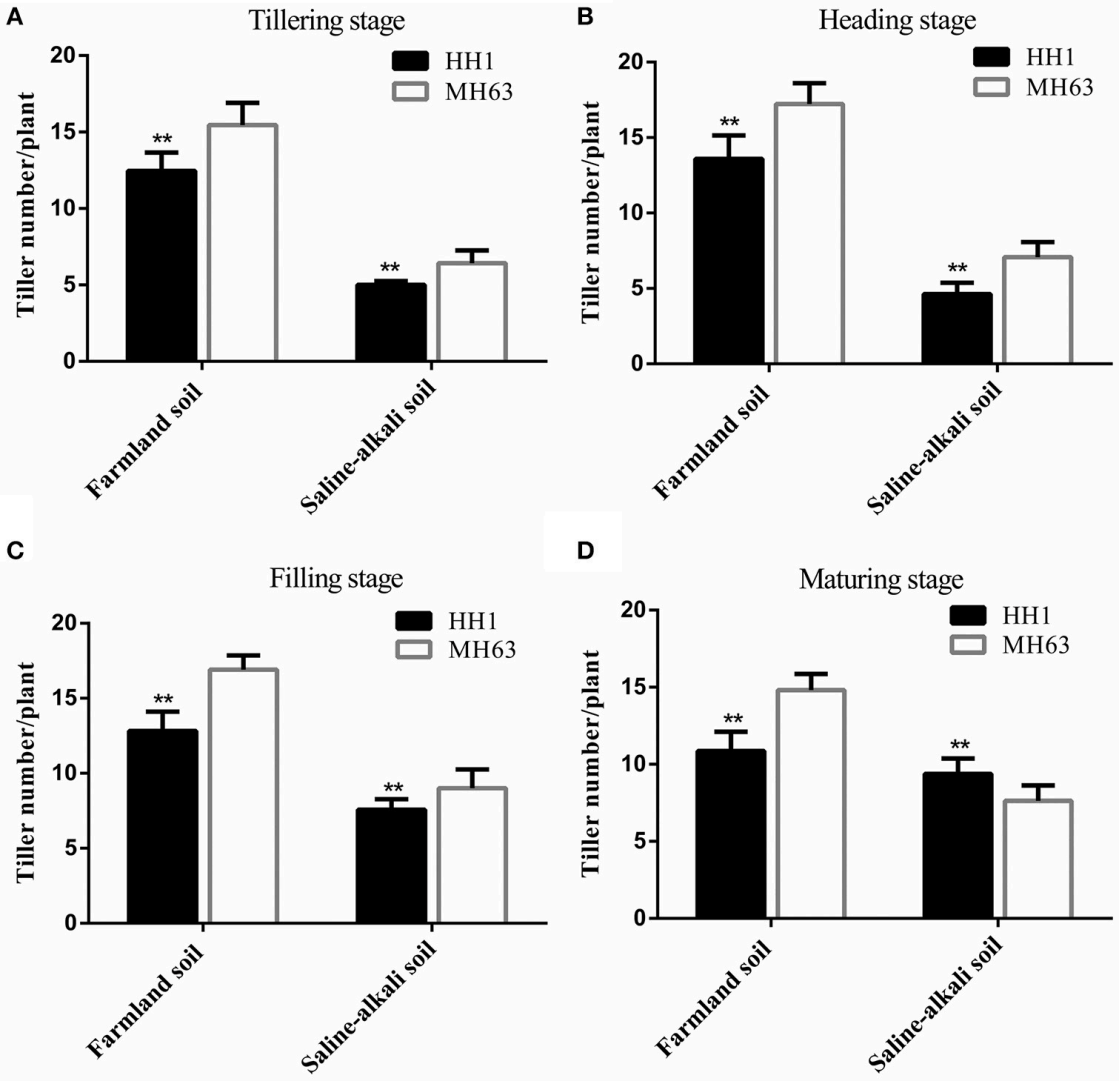
The effective tiller number per plant (mean ± SEM) of HH1 and MH63 rice grown on simulated farmland and saline-alkaline soil at four stages: **(A)** tilling stage, **(B)** heading stage, **(C)** filling stage, and **(D)** maturing stage. Farmland soil values with ** were significantly different from those of saline-alkaline soil according to the t-test (*p* < 0.01).

As shown in Figure 3, there were significant differences in the effective tiller number between HH1 and MH63 rice under the two soil conditions (*p* < 0.01). The effective tiller number in the same rice line was significantly higher for plants grown on farmland soil than in those grown on saline-alkaline soil by ~1.16–2.94 fold at the four key growth stages.

Under farmland soil conditions, the effective tiller number of both HH1 and MH63 rice was similar throughout the growth period. It initially increased, and then decreased with growth. The effective tiller number of HH1 rice was significantly lower than that of MH63 rice by ~19–27% at the tillering, heading, filling, and maturing stages (*p* < 0.01). On saline-alkaline soil, the effective tiller number of HH1 and MH63 rice were significantly different from those of plants grown on farmland soil throughout the growth stage. The effective tiller number in HH1 rice was significantly lower than that for MH63 rice by ~22–35% at tillering, heading, and filling (*p* < 0.01). The effective tiller number of HH1 rice was significantly higher than that of MH63 rice at the maturing stage. There was a significant fitness cost between HH1 and MH63 rice in terms of the effective tiller number grown on saline-alkaline soil. However, the magnitude of this fitness cost was relatively higher than that of the rice grown on farmland soil (Table 3). The three-way ANOVA indicated that the soil, exogenous gene, growth stage, interaction between exogenous gene and soil, interaction between soil and growth stage, interaction between exogenous gene and growth stage, and interaction among the three factors significantly affected the effective tiller number (Table S2).

#### Biomass

**Figure 4:**
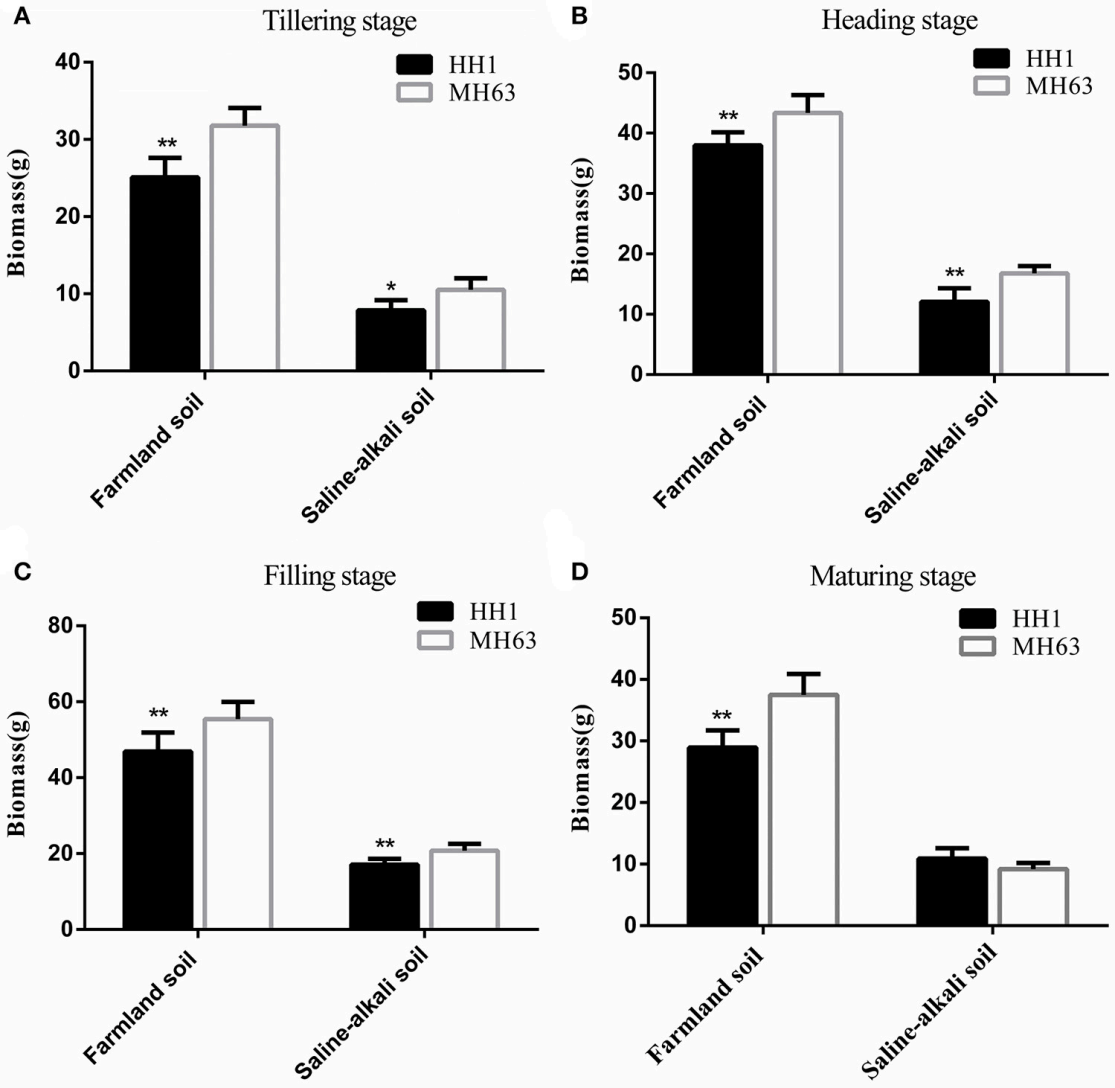
Biomass (mean ± SEM) of HH1 and MH63 rice grown on simulated farmland and saline-alkaline soil at four stages: **(A)** tilling stage, **(B)** heading stage, **(C)** filling stage, **(D)** maturing stage. Values for HH1 rice with * and ** were significantly different from those of MH63 rice according to the *t*-test (*p* < 0.05 and *p* < 0.01, respectively).

As shown in Figure 4, there were significant differences in biomass between HH1 and MH63 rice under the two soil conditions (*p* < 0.01). The biomass of the same rice line was significantly higher in those grown on farmland soil than in those grown on saline-alkaline soil by ~2.43–4.22 fold at the four key growth stages.

For plants grown on farmland soil, the changes in biomass in both HH1 and MH63 rice were similar throughout the = growth stage. It initially increased, and then decreased with growth. The biomass of HH1 rice was significantly lower than that of MH63 rice by ~15–25% at the tillering, heading, filling, and maturing stages (*p* < 0.05). On saline-alkali soil, the biomass of HH1 rice was significantly lower than that of MH63 by ~18–35% at the tillering, heading, and filling stages (*p* < 0.05). There was a significant fitness cost between HH1 and MH63 rice in terms of biomass when grown on saline-alkaline soil. The magnitude of this fitness cost was relatively higher than that of the rice grown on farmland soil (Table 3). The three-way ANOVA indicated that the soil, exogenous gene, growth stage, and interaction between exogenous gene and soil, interaction between soil and growth stage significantly affected biomass (Table S2).

### Effect of transgene expression on reproductive growth indices of transgenic rice HH1 under saline-alkali soil

As shown in Table 2, there were significant differences in yield-related traits between HH1 and MH63 rice under the two soil conditions (*p* < 0.01). The yield-related traits of the same rice line were significantly higher in plants grown on farmland soil than in those grown on saline-alkaline soil.

**Table 2:** Reproductive traits of HH1 rice and MH63 rice grown on farmland and saline-alkaline soils.

**Reproductive trait**	**Farmland soil**			**Saline-alkaline soil**					
	**HH1**	**MH63**	**P**	**HH1**	**MH63**	***P***
Effective panicle number/plant	7.67 ± 0.47**	10.33 ± 0.94	0.00	3.00 ± 0.00	3.00 ± 0.00	
Panicle length (cm)	27.23 ± 0.76	26.72 ± 1.55	0.47	13.69 ± 1.32	13.28 ± 2.62	0.96
Panicle weight (g)	25.32 ± 0.88**	28.42 ± 1.43	0.00	0.52 ± 0.10**	1.30 ± 0.18	0.00
Filled grain number/plant	683.00 ± 54.06**	878.00 ± 32.07	0.00	6.50 ± 2.43**	14.50 ± 3.04	0.00
Filled grain weight/plant (g)	19.71 ± 1.56**	23.42 ± 1.12	0.00	0.16 ± 0.06**	0.36 ± 0.09	0.00
Total grain number/plant	831.67 ± 40.12**	*1, 214.00*±90.23	0.37	124.67 ± 18.00**	291.67 ± 20.40	0.00
Filled grain number/panicle	89.08 ± 4.34	85.58 ± 7.14	0.00	2.17 ± 0.81**	4.83 ± 1.01	0.00
1,000 grain weight (g)	27.66 ± 0.55	27.84 ± 0.38	0.56	25.77 ± 0.41	25.86 ± 0.57	0.80
Seed-setting rate (%)	82.36%±7.83%*	72.66%±5.19%	0.04	5.00%±2.26%	5.00%±1.00%	0.65

Mean ± SEM followed by HH1 rice with ^*^ and ^**^ were significantly different from MH63 rice according to the t-test (p < 0.05 and p < 0.01, respectively).

The seed-setting rate for HH1 rice grown on farmland soil was significantly higher than that of MH63 rice. In contrast, on the same soil, the effective panicle number, panicle weight, filled grain number per plant, total grain number per plant, and filled grain weight per plant of HH1 were significantly lower than those of MH63 rice by ~25.75, 15.84, 22.21, 31.49, and 7.00%, respectively (*p* < 0.01). In the plants grown on saline-alkaline soil, the panicle weight, filled grain number per plant, total grain number per plant, filled grain weight per plant, and filled grain number per panicle of HH1 rice were significantly lower than those of MH63 rice by ~60.00, 55.17, 55.56, 57.26, and 55.07%, respectively (*p* < 0.01). Nevertheless, HH1 and MH63 rice showed similar performances in terms of effective panicle number, panicle length, 1000-grain weight, and seed-setting rate. There were significant fitness costs in most yield-related traits between HH1 and MH63 rice grown on saline-alkaline soil. The magnitude of these fitness costs was relatively higher than those between HH1 and MH63 rice grown on farmland soil (Table 3).

**Table 3:** Fitness for vegetative and reproductive traits of HH1 vs. MH63 rice grown on farmland and saline-alkali soils.

			**Farmland soil**	**Saline-alkali soil**
Vegetative trait	Fitness	Plant height (cm)	1.08*	1.06*
		Tiller number	0.78*	0.75*
		Biomass(g)	0.83*	0.76*
	Total fitness		0.90*	0.86*
Reproductive trait	Fitness	Effective panicle number per plant	0.74*	1.00
		Panicle length (cm)	1.02	1.03
		Panicle weight (g)	0.89*	0.39*
		Filled grain number per plant	0.78*	0.45*
		Grain weight per plant (g)	0.84*	0.44*
		Grain number per plant	0.69*	0.43*
		Filled grain number per panicle	1.04	0.45*
		Thousand grain weight (g)	0.99	0.99
		Seed-setting rate (%)	1.13*	1.00
	Total fitness		0.90*	0.69*

*Fitness ratio was defined as agronomic traits of HH1 vs. MH63 rice; Total fitness was defined by the average value of all fitness for vegetative traits or reproductive traits*.

* *indicates fitness significantly more than or less than 1.00 according to t-test (p < 0.05)*.

The two-way ANOVA indicated that soil conditions significantly affected all yield-related traits. Exogenous protein level significantly negatively influenced most yield-related traits with the exceptions of spike length and 1,000-grain weight. The interaction between these two factors was generally higher than that of other pairwise combinations (Table S3).

## Discussion

In the present study, the *cry1Ab/c* transgenic HH1 rice was cultivated in environments with low target insect pressure. The expression pattern of exogenous Cry1Ab/c protein and the fitness performance of HH1 rice during vegetative and reproductive growth stages were evaluated on simulated farmland and saline-alkaline soils. The results indicated that the ecological risk of *cry1Ab/c* transgenic rice as determined by vegetative and reproductive growth indices is not expected to be higher than that of the parental rice Minghui63, if the former escapes into regions with natural saline-alkaline soil.

### Expression of exogenous cry1Ab/c protein

Environmental conditions significantly affect the expression of exogenous proteins in *Bt* transgenic crops (Burke and Rieseberg, 2003; Chen et al., 2018b). Our results indicated that the expression of exogenous Cry1Ab/c protein in transgenic HH1 varied temporally and spatially in rice grown on saline-alkaline and farmland soils. The aforementioned findings corroborate with those of other studies in which some insect-resistant *Bt* transgenic rice lines (Jiang et al., 2016; Wang et al., 2016; Zhang et al., 2016), cotton lines (Olsen et al., 2005; Siebert et al., 2009; Chen et al., 2012), and corn lines (Badea et al., 2010) presented temporal and spatial changes in the exogenous Bt protein expression level under natural insect pressure in field. In saline-alkaline soil, the average expression level of exogenous Cry1Ab/c protein in the leaves and stems of HH1 rice was significantly lower than that of plants grown on farmland soil throughout the growth stage. Therefore, the expression of exogenous protein Cry1Ab/c in HH1 rice was significantly inhibited by saline-alkali soil. Similar studies have shown that the expression level of exogenous protein in insect-resistant *Bt* transgenic corn lines under high temperature, low temperature, drought, and flooding stress conditions was significantly lower than those for plants grown under normal, stress-free growth conditions (Traore et al., 2000; Trtikova et al., 2015). The expression level of exogenous protein in insect-resistant *Bt* tansgenic cotton lines under salt (Jiang et al., 2006; Luo et al., 2017), flooding (Luo et al., 2008), and low-temperature stress conditions (Addison and Rogers, 2010) was significantly lower than that in plants grown under normal, stress-free conditions. Taken together, the findings of the present and previous studies suggest that external stress conditions might reduce the expression of exogenous Bt protein in transgenic crops.

The results showed that the expression of Cry1Ab/c protein in HH1 rice grown on saline-alkaline soil was lower than that of the same plants grown on farmland soil. However, there was still a considerably high level of Cry1Ab/c protein expression in the plants on saline-alkaline soil. Therefore, HH1 rice could still effectively resist target insect even on saline-alkaline soil. Although HH1 was grown on saline-alkali soil under low target insect pressure, it has comparatively low insect resistance, due to the biosynthesis of Cry1Ab/c protein for which considerable amounts of material and energy must be consumed from the host cell. This will avoid fitness benefit toward vegetative and reproductive growth abilities of HH1 rice.

### Vegetative growth of *cry1Ab/c* transgenic rice under two soil conditions

The results of the present study indicated that plant height, number of tillers, and biomass of HH1 and MH63 rice were significantly lower in plants grown on saline-alkaline soil than in those grown on farmland soil (*P* < 0.01). These findings were consistent with those of other studies in which some insect-resistant *Bt* transgenic cotton lines under salt and flooding stresses (Jiang et al., 2006; Kaur et al., 2015) and drought stress (Zhang et al., 2017), and some insect-resistant *Bt* transgenic corn lines under drought stress (Traore et al., 2000) had significantly lower vegetative growth ability than that of plants grown under normal, stress-free conditions. In saline-alkaline and farmland soils without target insect pressure, only the plant height of HH1 rice was significantly higher than that of MH63 rice at most growth and development stages. In contrast, the vegetative growth indices, such as effective tiller number, biomass, and others of HH1 were significantly lower than those of MH63 rice. Therefore, the fitness cost was significant. Previous studies have shown that the height of transgenic *Bt/CpT1* rice was significantly higher than that of the parental rice whereas the tiller numbers of the transgenic rice was significantly lower than those of the parental strain when the plants were grown under a glasshouse with extremely low insect pressure (Chen et al., 2006). The biomass of transgenic *cry2A* rice was significantly lower than that of the parental rice under a field condition with low insect pressure (Jiang et al., 2013b). Furthermore, studies have also compared the underlying fitness cost of insect-resistant *Bt* transgenic rice lines with that of the parental rice line in terms of vegetative growth indices, such as plant height and root length, under field condition with low insect pressure (Shu et al., 2002; Kim et al., 2008). The results have revealed that the exogenous *Bt* gene confers a vegetative growth disadvantage to transgenic rice under low insect pressure. The results also indicated that under both soil conditions, a lack of target insect pressure incurs significantly higher fitness cost for vegetative growth in HH1 rice than in MH63 rice. The difference in fitness cost for vegetative growth between HH1 and MH63 was higher on saline-alkaline soil than on farmland soil. A possible explanation is that HH1 rice wastes metabolic energy and materials to biosynthesize Bt protein rather than allocating those resources toward the formation of vegetative tissues and incurring a significant fitness cost in the absence of target insect pressure (Zeller et al., 2010). The expression of Cry1Ab/c in plants grown on saline-alkaline soil was significantly lower than that in plants grown on farmland soil. Nevertheless, its expression level remained relatively high in HH1 rice, resulting in a comparatively higher fitness cost. The aforementioned findings corroborated those of previous reports in which insect-resistant *cry1Ab* transgenic rice grown under simulated semi-wild growth conditions with naturally low insect pressure (Su et al., 2013) and those grown under natural growth conditions with naturally high insect pressure (Liu et al., 2015) could present lower vegetative growth rates than those of parental rice lines.

### Reproductive growth of *Cry1Ab/c* transgenic rice under two soil conditions

The results of the present study indicated that the reproductive growth indices of HH1 and MH63 rice in saline-alkaline soil were significantly lower than those of plants in farmland soil (*P* < 0.01). These observations were consistent with those reported by other studies in which some insect-resistant *Bt* transgenic cotton lines (Zhang et al., 2017) and insect-resistant *Bt* transgenic corn lines (Brewer et al., 2014) under soil water stress presented significantly lower reproductive growth indices than those of the same plants grown under normal, stress-free growth conditions. On saline-alkaline and farmland soils in the absence of target insect pressure, filled grain number per plant, filled grain weight per plant and other important reproductive indices of HH1 rice were significantly lower than that of MH63 rice under the same soil conditions. Therefore, there was a significant fitness cost. Previous studies have showed that reproductive growth indices, such as filled grain number per plant, filled grain weight per plant, effective panicle number, and others of transgenic *Bt/CpT1* rice were significantly lower than those of the parental MH86 rice grown under both greenhouse and field conditions with very low insect pressure (Chen et al., 2006; Xia et al., 2010). Significant fitness costs reflected in various reproductive indices, such as filled grain number per spike, seed-setting rate, total grain weight or yield, and 1000- grain weight, have been reported by previous studies in transgenic *cry1Ac* rice (Shu et al., 2002), *cry1Ab* rice (Kim et al., 2008), and *cry1Ab/c* rice (Xia et al., 2011) under field conditions with natural low insect pressure. These results suggest that the exogenous *Bt* gene conferred substantial reproductive growth disadvantage to transgenic *Bt* rice under low insect pressure. The results also indicated that in both soil types, the lack of target insect pressure incurs a significant fitness cost in terms of reproductive growth ability in HH1 rice relative to MH63 rice. Moreover, the reproductive growth fitness cost difference between HH1 and MH63 rice grown on saline-alkaline soil was higher than that of the same strains grown on farmland soil. A possible explanation is that under low target insect pressure, HH1 had significantly lower vegetative growth (tiller numbers, biomass, and other parameters) than that of MH63 during the main growth and development stages. Therefore, HH1 incurred higher fitness cost than that of MH63 rice. If transgenic *cry1Ab/c* rice HH1 escapes from the tillage system into the natural saline-alkaline ecosystem, it might have weaker vegetative and reproductive growth abilities than those of parental MH63. Therefore, it is unlikely that HH1 might establish its own population to disperse or proliferate in natural saline-alkaline ecosystems in the event of its escape from the tillage system where it originated. Other studies have reported that insect-resistant *cry1Ab* transgenic rice in simulated semi-wild growth conditions and natural low insect pressure (Su et al., 2013), insect-resistant *cry1Ab/c* transgenic rice in simulated natural growth conditions and natural high insect pressure (Liu et al., 2015), and insect-resistant *Bt* transgenic cotton in simulated natural growth conditions and natural high insect pressure (Eastick and Hearnden, 2006) are at risk of having lower reproductive ability than their parental strains.

In conclusion, this study demonstrated that, under the saline-alkaline and farmland soil conditions, *cry1Ab/c* transgenic HH1 rice has weaker vegetative and reproductive growth abilities than those of its parental MH63 rice. HH1 also presented significantly high fitness costs especially under saline-alkaline soil conditions. In the latter case, the elevated fitness cost correlated with the consumption of material and energy required for the expression of exogenous Cry1Ab/c protein. Therefore, if *cry1Ab/c* transgenic HH1 rice escapes from the tillage system into the natural saline-alkaline ecosystem, it would be less likely to proliferate there than its parental MH63 rice. Consequently, the ecological risk is extremely low. However, these conclusions were drawn from the data obtained using simulated saline-alkaline conditions with the absence of target insect pressure. Nevertheless, under the natural saline-alkaline environment, the *cry1Ab/c* transgenic rice could still encounter target insect pressures significantly lower than those in the farmland environment. Therefore, in the future, other habitat conditions, including natural saline-alkaline, target insect, weed competition will be simulated to assess the fitness effects of *Bt* transgenic rice under these conditions. The natural ecological risk of *Bt* transgenic rice could thus be evaluated more accurately, providing ample data to use in the management of environmental risks of *Bt* transgenic crops.

## Author contributions

JF and BL designed and carried out the experiments and wrote the manuscript. YS helped analyze the data. WS, ZF, LZ, and XS helped calibrate the manuscript.

### Conflict of interest statement

The authors declare that the research was conducted in the absence of any commercial or financial relationships that could be construed as a potential conflict of interest.
