# Supplementary material for: Fitness Cost of Transgenic cry1Ab/c Rice Under Saline-Alkaline Soil Condition
Source: Front Plant Sci. 2018 Oct 23;9:1552. doi: 10.3389/fpls.2018.01552 (PMC6206443; doi:10.3389/fpls.2018.01552)
Supplement: Supplementary file 1 [file Data_Sheet_1.pdf]

Supplementary information

Table S1

Three-way ANOVA analysis the effects of soil conditions, growth stages, tissues, and interactions among them on Cry1Ab/c protein expression in transgenic HH1 rice

	Cry1Ab/c protein (μg g^{-1} FW)		
	df	F	*P*
Soil	1	45.50	0.00
Growth Stage	4	36.46	0.00
Tissue	1	332.14	0.00
Soil × Growth Stage	4	2.00	NS
Soil × Tissue	1	5.66	0.02
Growth Stage × Tissue	4	11.15	0.00
Soil × Growth Stage × Tissue	4	1.91	NS

$p < 0.05$ indicates significant difference; NS indicates no significant difference

Table S2

Three-way ANOVA analysis the effects of soil conditions, rice lines and growth stages on vegetative growth indices

	Plant height (cm)			Tiller number			Biomass (g)		
	df	F	P	df	F	P	df	F	P
S	1	3,560.86	0.00	1	3,057.84	0.00	1	2,019.01	0.00
Rl	1	153.65	0.00	1	304.79	0.00	1	77.42	0.00
Gs	3	315.70	0.00	3	34.92	0.00	2	161.18	0.00
S×Rl	1	13.46	0.00	1	116.25	0.00	1	21.61	0.00
S ×Gs	3	134.11	0.00	3	70.29	0.00	2	23.40	0.00
Rl×Gs	3	11.51	0.00	3	8.82	0.00	2	0.57	NS
S×Rl×Gs	3	1.10	NS	3	11.99	0.00	2	3.87	NS

S: Soils, Rl: Rice lines, Gs: Growth stages. $p < 0.05$ indicates significant difference; NS indicates no significant difference.

Table S3

Two-way ANOVA analysis the effects of soil conditions and rice lines on reproductive growth indices

Reproductive traits	Soil			Rice line			Soil × Rice line		
	df	F	*P*	df	F	*P*	df	F	*P*
Effective panicle number/plant	1	648.00	0.00	1	32.00	0.00	1	32.00	0.00
Panicle length (cm)	1	1,088.51	0.00	1	0.53	NS	1	0.27	NS
Panicle weight (g)	1	4,044.05	0.00	1	22.50	0.00	1	8.10	0.00
Filled grain number/plant	1	3,123.95	0.00	1	54.28	0.00	1	42.06	0.00
Grain weight/plant (g)	1	2,722.35	0.00	1	22.87	0.00	1	18.50	0.00
Grain number/plant	1	2,130.80	0.00	1	75.99	0.00	1	73.90	0.00
Filled grain number/panicle	1	360.54	0.00	1	165.02	0.00	1	179.47	0.00
Thousand grain weight (g)	1	79.49	0.00	1	0.37	NS	1	0.05	NS
Seed-setting rate (%)	1	1,111.24	0.00	1	5.67	0.03	1	4.62	0.04

$p < 0.05$ indicates significant difference; NS indicates no significant difference
